# Background and common lesions in the female reproductive organs of giant anteaters (*Myrmecophaga tridactyla*)

**DOI:** 10.3389/fvets.2024.1287872

**Published:** 2024-01-24

**Authors:** Fernanda Barthelson Carvalho de Moura, Zara Alves Lacerda, José Luiz Catão-Dias, Pedro Enrique Navas-Suárez, Karin Werther, Sarah Raquel Jesus Santos Simões, Renato de Lima Santos, Daniel Felipe Barrantes Murillo, Tatiane Terumi Negrão Watanabe, Carlos Eduardo Fonseca-Alves, Noeme Sousa Rocha

**Affiliations:** ^1^School of Veterinary Medicine and Animal Science, São Paulo State University (UNESP), São Paulo, Brazil; ^2^School of Veterinary Medicine and Animal Science, University of São Paulo (USP), São Paulo, Brazil; ^3^Veterinary Medicine Program, University Center FAM, São Paulo, Brazil; ^4^School of Agricultural and Veterinarian Sciences, São Paulo State University (UNESP), São Paulo, Brazil; ^5^Veterinary School, “Universidade Federal de Minas Gerais” (UFMG), Belo Horizonte, Brazil; ^6^College of Veterinary Medicine, Auburn University, Auburn, AL, United States; ^7^Antech Diagnostics, Los Angeles, CA, United States; ^8^Institute of Health Sciences, Paulista University (UNIP), São Paulo, Brazil

**Keywords:** female, reproduction, morphology, histopathology, xenarthra

## Abstract

The giant anteater (*Myrmecophaga tridactyla*) is a vulnerable species in South America and is considered endangered or near extinction in Central America. Therefore, studies describing the reproductive characteristics of this species are pivotal for its conservation. Thus, this study aimed to provide a morphological description of the female reproductive tissues of this species. We collected tissue samples from six female giant anteaters and performed gross, morphological, and histochemical analyses. Five adult subjects and one juvenile were included in the study. In the ovary, classifications were made according to the follicle and oocyte sizes: primordial, primary, secondary, early antral, or antral. Typical follicles with a single oocyte surrounded by a simple or stratified layer of cubic epithelium, atretic follicles, corpora lutea, corpora albicans, and ovarian cysts were also observed. No ovarian lesions were observed. By contrast, endometritis, metritis, mucometra, and endometrial cysts were identified in the uterus. Uterine alterations in these subjects were frequent and could affect reproduction.

## 1 Introduction

Xenarthra is a superorder of placental mammals endemic to the American Continent, including the orders Pilosa (anteaters and sloths) and Cingulata (armadillos). The giant anteater (*Myrmecophaga tridactyla*) is a member of the Myrmecophagidae family. According to the International Union for Conservation of Nature (IUCN) Red List of Threatened Species, giant anteaters are considered to be a vulnerable or extinct species in different Latin American countries (1). Several factors, such as predatory hunting, forest burning, and blunt trauma secondary to wildlife–vehicle collisions, contribute to the reduction in the giant anteater population (1–7). The lack of governmental programs for conservation (8), slow reproductive cycles in nature (long gestation periods, birth of only one cub per year, and extended parental care) (2), and difficulties with captive breeding programs are factors that influence species preservation (9). The population of giant anteaters has been increasingly affected by vehicle collisions. Studies have shown that death rates are higher in males than in females among giant anteaters living near roads (within <2 km) (10, 11).

The scarcity of studies enhancing reproductive understanding and of available reproductive protocols and biotechnologies also contributes to challenges in reproduction (9). Morphological and histopathological descriptions of the reproductive tracts of this species are highly relevant in determining the typical features or recognizing clinically significant lesions in these organs, which may interfere with individual fertility. The lack of knowledge in this area can hinder conservation work if normal and abnormal findings in the sexual organs are poorly defined. A recent study has identified the structural and ultrastructural morphology of the prostate gland in giant anteaters and revealed its histological and immunohistochemical features (12). Another study has described the morphology and histology of the reproductive tracts (13).

Different lesions and female reproductive disorders have been described in several domestic and wild species, including lesser anteaters (*Tamandua tetradactyla*) (14–17). To our knowledge, there have been few studies that have investigated and analyzed the histochemical features and common lesion alterations in giant female anteaters (18). Histopathological characterization of these alterations is important for an understanding of individual fertility. Thus, this study aimed to generate new knowledge regarding pathologies affecting the ovaries and uterus of giant anteaters to improve the diagnosis of reproductive disorders in this charismatic species.

## 2 Materials and methods

### 2.1 Ethical approval

This study was approved by three Brazilian committees responsible for conducting wildlife research: the System for Genetic Heritage and Associated Traditional Knowledge (#C1018E9), the Chico Mendes Institute for Biodiversity Conservation (#7685–1), and São Paulo State University Committee on the Use of Animals in Research (177/2020).

### 2.2 Sample collection

Six female giant anteaters were included after being received at our institutional wildlife center within a maximum interval of 12-h after death due to blunt trauma force (Table 1). Their ages were determined based on their body weights according to the existing literature (13). Representative sections of both the ovaries and uterus were systematically collected and fixed in neutral-buffered 10% formalin during necropsy. Samples were collected at São Paulo State University (UNESP, Brazil), the University of São Paulo (USP, Brazil), and the Universidade Federal de Minas Gerais (UFMG, Brazil) between January 2000 and December 2021.

**Table 1:** Giant anteater autopsied information.

Identification	Age	Weight	Background lesions/Microscopic findings	Cause of death
Subject 1	Juvenile	N/E	Moderate mucometra and prominent corpora lutea	Wildlife–vehicle collision leading to death due to blunt trauma force
Subject 2	Juvenile	N/E	Mild chronic lymphoplasmacytic endometritis and mucometra	
Subject 3	Adult	26 kg	Multiple follicular ovarian cysts	
Subject 4	Adult	27 kg	Moderate acute neutrophilic endometritis and mucometra	
Subject 5	Adult	N/E	Mild chronic lymphoplasmacytic edematous endometritis and mucometra	
Subject 6	Adult	N/E	Mild acute neutrophilic metritis and mucometra	

### 2.3 Histochemistry

For the histochemical analysis, tissue samples were fixed in 10% buffered formalin and embedded in paraffin. Afterward, 4-μM-thick tissue sections were stained with hematoxylin and eosin for morphological evaluation. Periodic acid–Schiff (PAS) and Masson’s trichrome staining were performed to evaluate structural findings.

### 2.4 Morphological and histological analysis

Ovarian and uterine morphological analyses were performed according to the methods established by Rossi et al. (17) and Fromme et al. (13). The evaluation was performed in a double-blind fashion: one at São Paulo State University (N.S.R.) and the other at North Carolina State University (T.T.N.W.). A qualitative analysis was conducted instead of a descriptive analysis due to the small number of animals included. Lesions were described to determine the frequency of each type of lesion.

## 3 Results

### 3.1 Gross evaluation

**Figure 1:**
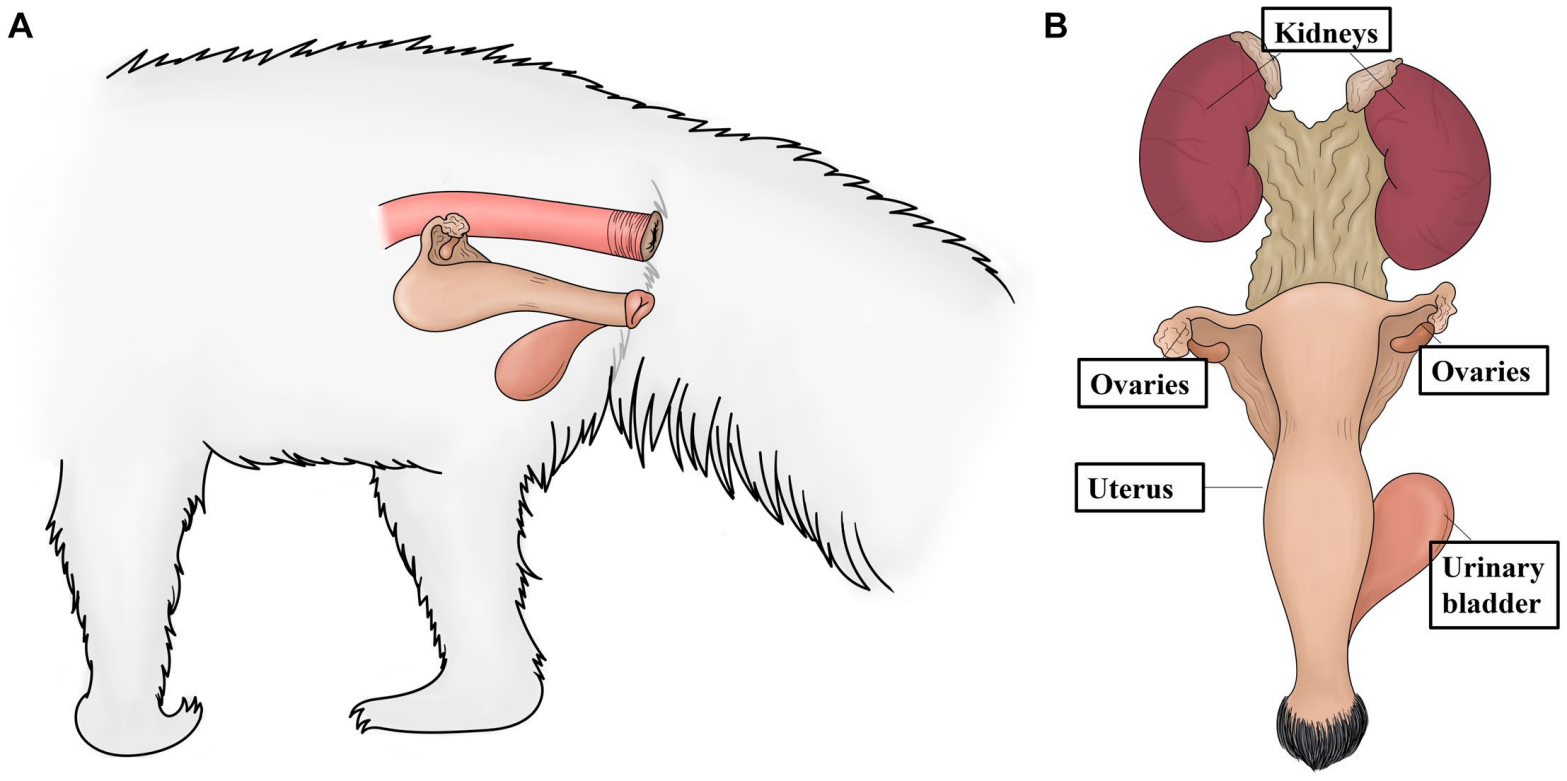
Schematic representation of the anatomic localization of the ovaries and uterus of the giant anteater. **(A)** Localization of the ovaries and uterus dorsal to the urinary bladder (and ventral to the rectum). **(B)** The urogenital tract of a female giant anteater, showing the position of the ovaries and uterus cranial to the urinary bladder and caudal to the kidneys.

**Figure 2:**
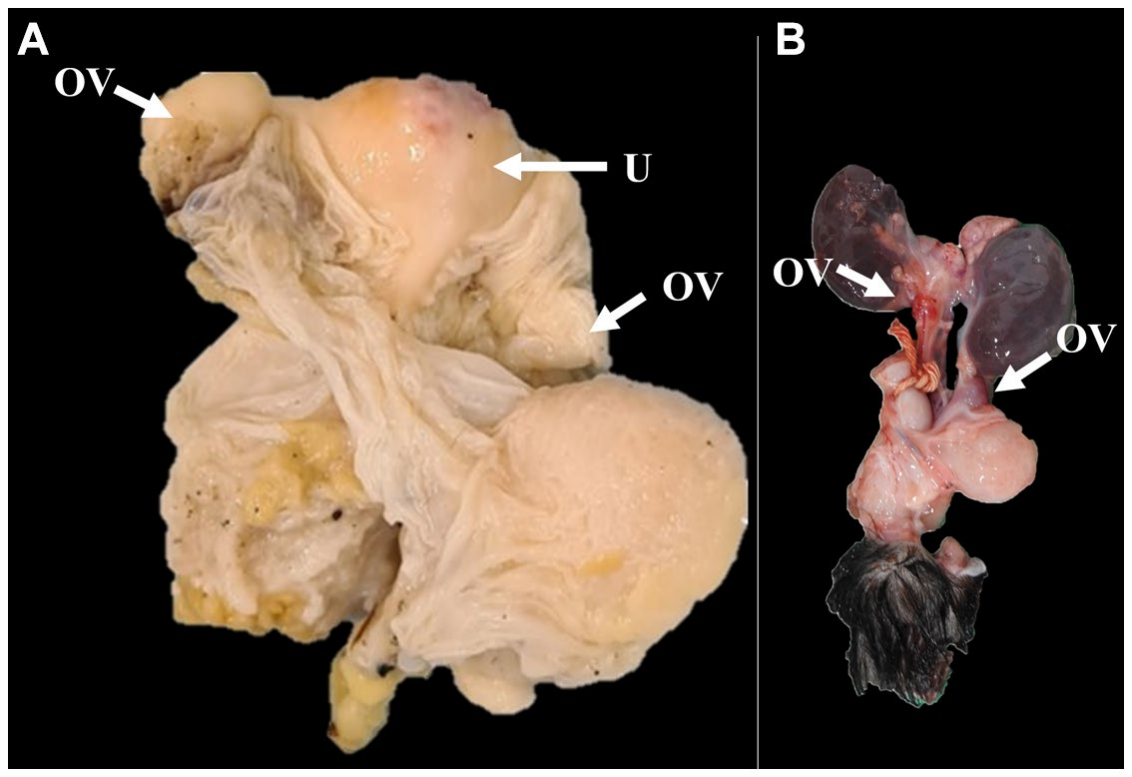
Gross images of the ovaries and uterus of adult **(A)** and juvenile **(B)** giant anteaters. **(A)** The formalin-fixed ovaries and the simple uteri are observed. **(B)** Macroscopic appearance of the ovaries of the giant anteater, ovoid in shape, located dorsal to the urinary bladder. OV: ovary; U: uterus.

The reproductive tracts of six free-living female giant anteaters were collected during routine postmortem examination. After the postmortem examination, ovary and uterine samples from five adult subjects (> 2 years) and one juvenile (< 2 years) were subjected to gross and microscopic assessment. There were no significant differences observed between tissues from subjects in these two different age categories. The ovaries showed an ovoid morphology and measured approximately 2 cm in length, 0.5 cm in width, and 0.5 in height. The simple uterine body was approximately 3 cm in length, 2.5 cm in width, and 2 cm in height. The ovaries and uterus were located dorsal to the urinary bladder and ventral to the rectum (Figures 1, 2).

### 3.2 Microscopic evaluation

Microscopically, the ovaries showed an architecture consisting of a simple cuboidal epithelium, ovaries, serosa, and dense connective tissue in the tunica albuginea. The ovarian parenchyma consisted of two zones. The cortical zone was located under the tunica albuginea and contained ovarian follicles at different stages of development (Figure 2). These stages were classified according to the follicle and oocyte sizes as primordial (mean diameter [follicle 88.24 μm; oocyte 47.2 μm; SD: 33.37 μm]; mean follicle number: 58), primary (mean diameter [follicle 125.02 μm; oocyte 38.72 μm; SD: 27.38 μm]; mean follicle number: 8), secondary (mean diameter [follicle 313.87 μm; oocyte 64.64 μm; SD: 176.23 μm]; mean follicle number: 4), early antral (mean diameter [follicle 953.68 μm; oocyte 152.27 μm; SD: 566.69 μm]; mean follicle number: 2), or antral (mean diameter [follicle 1806.89 μm; oocyte 374.42 μm; SD: 1012.28 μm]; mean follicle number: 1). The ovarian cortex surrounded the medullary zone, where the major vessels and nerves entered the ovary centrally and ramified to the periphery proximal to the follicles. We observed that the collagen fibers from the cortical and medullary zones were colored blue with Masson’s trichrome staining, showing rich collagen bunds in the uterine parenchyma, and that the zona pellucida and theca ovarian cells were positive for PAS staining.

**Figure 3:**
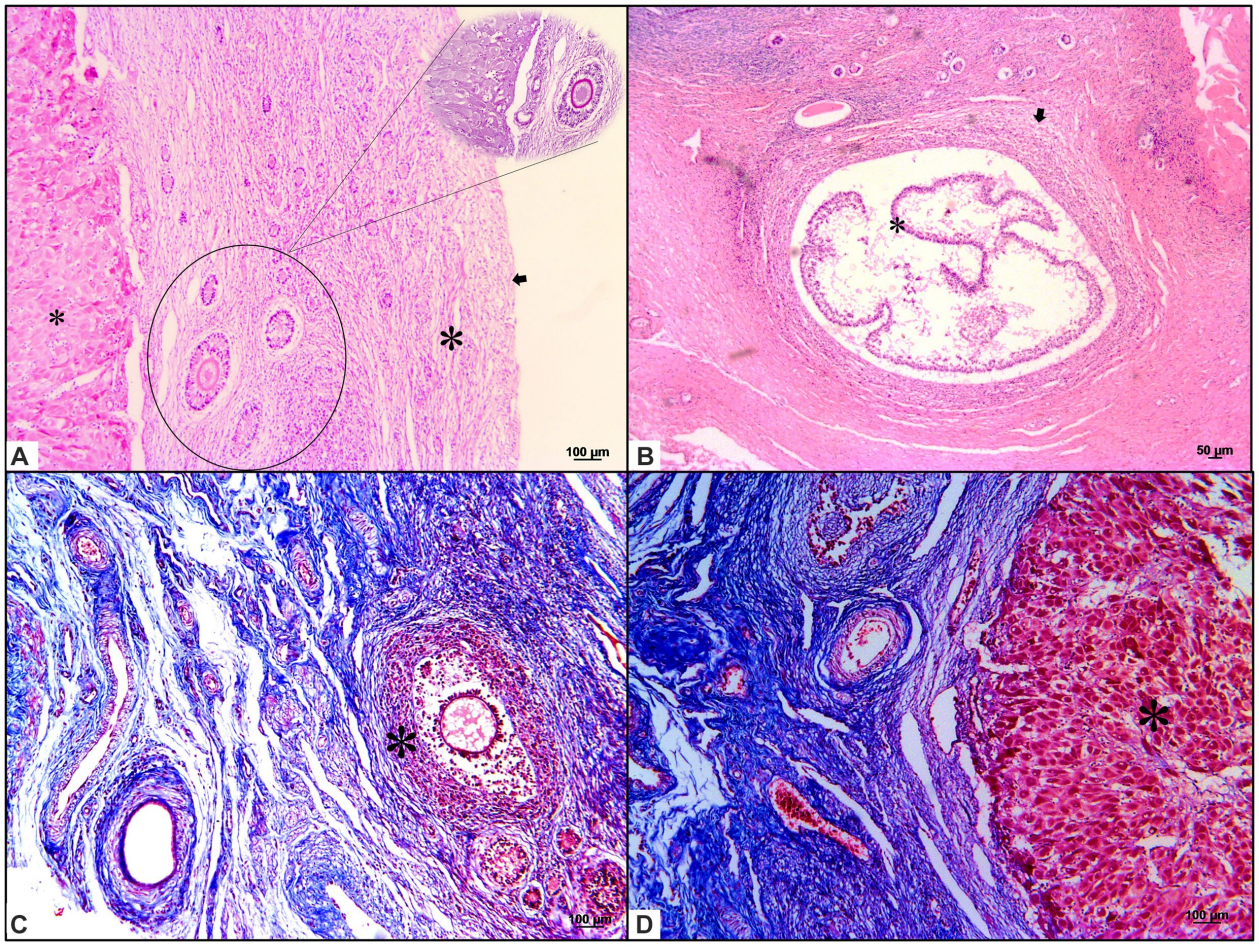
Histochemical characterization of background and common lesions in the ovaries of giant anteaters. **(A)** Ovaries with a simple cuboidal epithelium (small asterisk) on the serosa and dense connective tissue; the tunica albuginea (arrows), which is the ovarian parenchyma, is composed of two zones. The cortical zone (inside lined and non-lined circles, stained with hematoxylin–eosin and periodic acid–Schiff staining, respectively) is located under the tunica albuginea. Ovarian follicles in different stages of development are presented. The medullar zone (small asterisk), which is surrounded by the ovarian cortex (large asterisk), is where the major vessels and nerves enter the ovary centrally and ramify to the periphery proximal to the follicles. The ovarian serosa and parenchyma, stained with hematoxylin and eosin. **(B,C)** Typical follicles (arrows) with a single oocyte surrounded by a simple or stratified layer of cubical epithelium, atretic follicles, and ovarian cysts (large and small asterisks in **B,C**, respectively) were observed. Hematoxylin and eosin and Masson’s trichrome staining. **(D)** Medullary zone filled with a fibrous stroma; the presence of vessels and a corpora lutea (large asterisk) can be observed centrally. Masson’s trichrome staining.

Common lesions, such as typical atretic follicles containing degenerative granulosa cells and oocytes, were observed. Additionally, corpora lutea were visualized as centrally filled with the remains of the blood clot that formed after ovulation, surrounded by granulosa lutein cells with lutein cells on the outside. Some dense, round connective tissue organization, consisting of corpora albicans, was also identified in the cortical zone. The most typical background lesions were thin-walled ovarian cysts filled with pale acidophilic residual degenerative oocytes often surrounded by cell debris (Figure 3).

**Figure 4:**
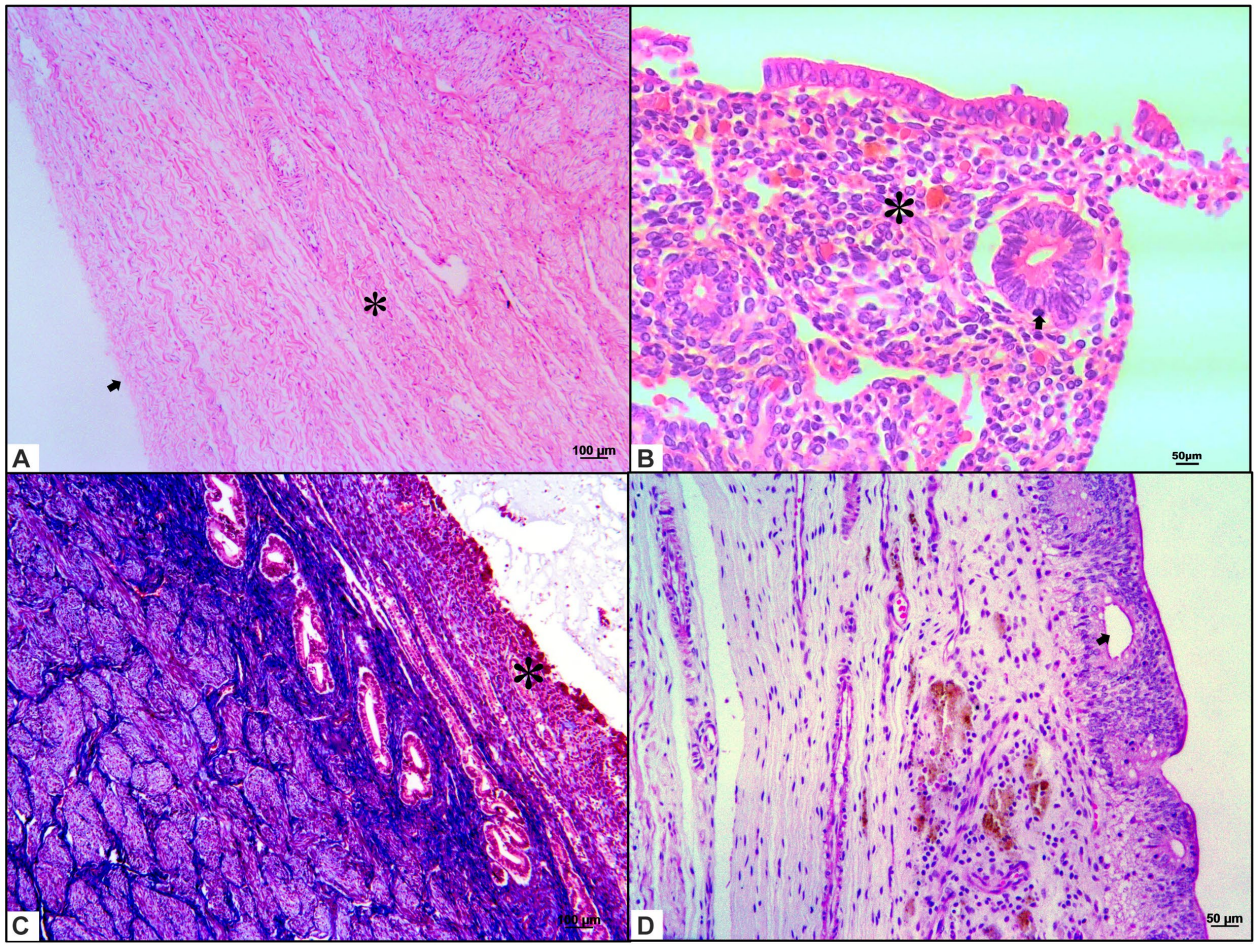
Histochemical characterization of background and common lesions in the uterus of giant anteaters. **(A)** The anteater uterus presents with three different layers. The outer layer, a perimetrium, is composed of loose connective tissues located peripherally (arrow). Second, the myometrium consists of one thin external longitudinal muscular layer and a second thick internal circular muscular layer (asterisk). Hematoxylin and eosin staining. **(B)** The simple cuboidal epithelium with folded endometrium near the uterine lumen presents as simple tubular glands surrounded by dense fibrous connective tissue (asterisk) with discrete leukocytic infiltration in uterine mucus (arrow). Hematoxylin and eosin staining. **(C,D)** Luminal secretion (asterisk, Masson’s trichrome staining in **C**) can be observed, and endometrial cysts (arrow, periodic acid–Schiff staining in **D**) disposed in the endometrial mucosa are accentuated.

Three different layers were observed in the uterine samples. First, the perimetrium was composed of loose connective tissues located peripherally. Second, the myometrium was found to consist of a thin external longitudinal layer and a thick internal circular muscular layer. The simple cuboidal epithelium with a folded endometrium near the uterine lumen presented with simple tubular glands surrounded by dense fibrous connective tissue. We observed that the collagen fibers from the perimetrium and muscle fibers from the myometrium were blue and red, respectively, after Masson’s trichrome staining (Figure 4C).

The most common lesion was a mucometra, which was characterized by a discrete uterine lumen dilated with fluid. The most commonly identified background lesions were endometritis and metritis, consisting of a uterine lumen moderately dilated with fluid and discrete leukocytic infiltration composed of mononuclear cells with the presence of lymphocytes and plasma cells in the endometrium and myometrium, respectively. One case showed endometrial cysts comprising thin-walled, round epithelial structures in the endometrium. Luminal secretions and epithelial cyst cells disposed of in the endometrial mucosa were positive for PAS staining (Figure 4).

## 4 Discussion

Studies investigating and analyzing the histochemical features of background and common lesions and alterations in female giant anteaters are rare (18). Histopathological characterization of reproductive abnormalities is important for an understanding of individual fertility. Our results contribute to the information available on ovarian and uterine lesions in giant anteaters through “ba/b = @histological analyses.

Previous studies have characterized the morphology and histology of the reproductive organs in giant anteaters (11, 12). Two studies involving female lesser anteaters (*Tamandua tetradactyla*) were performed. One other piece of research was conducted with the three-banded armadillo (*Tolypeutes matacus*), describing a case of uterine adenomyosis. A number of background and common ovarian lesions have been reported, including atretic follicles, corpora lutea, and corpora albicans (5, 17, 19).

We consider our results to be similar to the structural findings in female giant anteaters made in a previous study (13). Furthermore, the background and common reproductive lesions visualized have previously been described in humans and in domestic and wildlife species (14–16).

We observed that the giant anteater’s ovarian appearance is homologous with that observed in dogs. It is essential to mention that zone distribution occurs in both of these two species. Ovarian lesions have been studied to a greater extent in dogs, this being therefore a good model to better understand their pathogenesis in giant anteaters, which have been found to present features in common with dogs; this could allow us to infer that both species may develop similar lesions in the ovarian tissue. The follicles of giant anteaters were generally found to be larger than those of dogs. Nevertheless, oocyte size, granulosa cell number, and follicle diameter compared to other developmental follicular stages were homologs of those observed in dogs (20).

In giant anteaters and dogs, the atretic follicles have an irregular format, and granulosa cells have been observed in apoptosis; the follicular cysts present with thick layers of luteinizing granulosa cells, amid loose stroma; the luminal space is filled with eosinophilic material, and the corpora lutea have several vacuolated cells (20, 21).

The fallopian tubes could not be evaluated in this study because samples were not obtained during the research period. However, their structure in this species is already known. According to Fromme et al. (13), it is possible to histologically identify the infundibulum, ampulla, and isthmus, which are composed of mucosal, muscular, and serosal layers in giant anteaters. These three portions show variations in the lumen extension and width of the muscular layer, which become smaller and more significant as they become more distant from the ovaries and closer to the uterus, respectively (13).

We noted that the simple uterus in giant anteaters presented with extensive, well-marked fibrous tissue in the endometrium surrounding the tubular uterine glands. This feature is considered abnormal in domestic species, such as equines, interfering with an individual’s fertility (22, 23). Additionally, we observed that the myometrial layer was more prominent in this species than in domestic species or lesser anteaters (17, 24).

In giant anteaters and dogs, uterine background lesions have been found to consist of inflammatory infiltrates in different uterine layers, resulting in endometritis and metritis; moreover, luminal uterine secretion/edema is present, which may be associated with varying phases of the estrus cycle (19–21, 25–27).

The critical conservation status of giant anteaters significantly influences their ecological and evolutionary importance in the environment. As a species facing extinction in numerous countries, they are categorized as “vulnerable” by the IUCN, highlighting the urgency of conservation efforts (1, 28, 29). Thus, research involving the reproductive tract and fertility is fundamental to improving species conservation and protection and guaranteeing the maintenance of research programs involving these individuals. Another study has been conducted to evaluate the anatomical disposition of organs in the abdominal cavity in this species through computed tomography. All these complementary methods help to improve the detection of reproductive system lesions, contributing substantially to reproductive programs (30).

The results of the present study provide the first histochemical characterization of background lesions and common reproductive lesions in female giant anteaters. Overall, our results suggest that there is a degree of similarity between the ovarian and uterine pathophysiology of giant anteaters and that of other domestic species and humans. This information may help veterinary pathologists to report on reproductive alterations in female giant anteaters and allow them to characterize their impact on fertility.

## Data availability statement

The raw data supporting the conclusions of this article will be made available by the authors, without undue reservation.

## Ethics statement

This study was approved by three Brazilian committees responsible for conducting wildlife research: system for Genetic Heritage and Associated Traditional Knowledge (#C1018E9), Chico Mendes Institute for Biodiversity Conservation (#7685-1), and São Paulo State University Committee on the Use of Animals in Research (177/2020). The study was conducted in accordance with the local legislation and institutional requirements.

## Author contributions

FM: Conceptualization, Visualization, Writing – original draft, Writing – review & editing. ZL: Writing – review & editing. JC-D: Writing – review & editing. PN-S: Writing – review & editing. KW: Writing – review & editing. SS: Writing – review & editing. RS: Writing – review & editing. DM: Writing – review & editing. TW: Conceptualization, Visualization, Writing – review & editing. CF-A: Conceptualization, Visualization, Writing – original draft, Writing – review & editing. NR: Conceptualization, Visualization, Writing – original draft, Writing – review & editing.
